# Tabanidae and other Diptera on Camel’s Hump Vermont: Ecological Observations

**DOI:** 10.3897/zookeys.147.1989

**Published:** 2011-11-16

**Authors:** Jeffrey V. Freeman

**Affiliations:** 1 110 Gables Place, Rutland, VT 05701

**Keywords:** Tabanidae, Muscidae, Tachinidae, Camel’s Hump, polarized light, Vermont

## Abstract

A canopy trap and aerial nets led to finding 8 species of Tabanidae. There was an abundance of calyptrate muscoid flies. Camel’s Hump is in the Green Mountains of western New England, USA. Discovering Diptera on Camel’s Hump involved sixteen visits over 40 years. Upwards of 23 other Diptera species are listed. Habitats on the east side and above 762 m (2500 ft) elevation on Camel’s Hump differ from the west slope but the boreal forest on both sides is influenced by cloud and fog precipitation on trees. The cliffs just above the 900 m level along the east side are often overlooked, are not seen from the summit and provide access to morning sun for insects. Recent visits explored the role of polarized skylight in relation to the canopy trap, the boreal forest environment and flies found there.

## Introduction

Ross T. Bell introduced a group of students to the ecology of invertebrates on Camel’s Hump (Chittenden County, 44°19'N, 72°53'W) in 1972 and I was one of them. The purpose here is to bring together results of visits in 1972 (Freeman, 1973), 1998, 1999 and 2010 with use of nets and traps. I found eight species of tabanids but few individuals. Upwards of 15 species of calyptrate muscoid flies have been found in the large batches caught in a canopy trap. Officially Camel’s Hump (1244 m) from its summit down to 762 m is a natural area accessible only by four trails: Burrows Trail from the west, the Monroe Trail from the east and the Long Trail from north and south. It takes about two hours from a trail head to get on station and collecting for a day. The important question posed in our 1972 course was, what species of our chosen group are present on Camels Hump? Then, how do these invertebrates interact with their physical and biological environment on Camel’s Hump? More recently, how does the geomorphology of Camel’s Hump relate to these invertebrates? Also, what is the role of polarized skylight in the lives of the flies found here? Some aspects of human ecology enter this study as well.

After initial successful use of the canopy trap in 1970 and 1971 in studying Tabanidae it was a natural response to apply this method to Camel’s Hump in 1972. Pechuman (1971) set a record in trapping tabanids at Moose Bog east of Island Pond, VT, with 22 species of deer flies (*Chrysops*) and horse flies (*Hybomitra*). He used two canopy traps over a fifteen hour period and caught more than 10,000 tabanids. The main goal here was simply to collect flies, identify them and to look for significant patterns. Boone et al. (1992, 2000) made progress toward a checklist of Diptera and other invertebrates on Mt. Mansfield collecting at three stations, at 414 m, 610 m and 1170 m in their biodiversity survey on the west slope with weekly sampling with Malaise traps. On Camels Hump Sprague (1870) listed 21 species of Carabidae under Coleoptera but showed only 3 named species in the 8 genera of Diptera including *Pangonia* which is now *Stonemyia* in Tabanidae.

## Methods

Collecting with an aerial net was the usual method of collecting flies, and most often with sweeps but for whole morning or whole afternoon sessions the canopy trap was in operation. The Hut Clearing offered an open space in the boreal forest and was the junction of four trails. Net sweeps in the Hut Clearing especially but along trails as well and on the summit provided additional samples. Most labels show elevation. Another net made of nylon marquisette netting caught black flies and other very small Diptera. Wind can preclude use of the trap on the summit. With so many people there it is not advisable to leave the trap untended. The Hut Clearing is open to flies entering the clearing from four directions and above. It was possible to purchase dry ice in Burlington, wrap it in insulating material and have it on the trap in the Hut Clearing within three hours. Octenol (1-octen-3-ol, http://sigmaaldrich.com/united-states.html ), is a compound attractive to biting flies. In more recent years it was more convenient than dry ice.

The canopy trap was similar to that used by Pechuman (1981) and designed by Catts (1970). It reflects changes suggested by Townes (1962). Canopy traps catch tabanids readily, but performance can be improved with use of dry ice or octenol. (See Appendix No. 1) A Gressitt Malaise trap (Gressitt and Gressitt 1962) aided sampling tabanids at Monroe Base and is a flight interception trap.

The study area was in the area above 762 m elevation partly as studied by Siccama (1974), but his emphasis was the vegetation of the west slope of Camel’s Hump and other mountains in Vermont. Emphasis here has been mainly on the Hut Clearing at 1158 m, the summit, and along the Alpine Trail which goes from 0.48 km south of the summit at the Long Trail, crosses the Monroe Trail 0.48 km below the Hut Clearing, crosses exposed (Basque) ledges on the east side, and rejoins the Long Trail 1.28 km north of the Hut Clearing. The Alpine Trail passed a small sphagnum bog northeast of and below the Hut Clearing. Fig. 1 shows these locations. The Hut Clearing is immediately north of and about 86 m lower than the summit. The Little Bog is along the Alpine Trail near its north end. Detecting polarized skylight can be done with polarizing sunglasses but especially with the polarizing filter over the camera lens as in Fig. 2.

**Figure:**
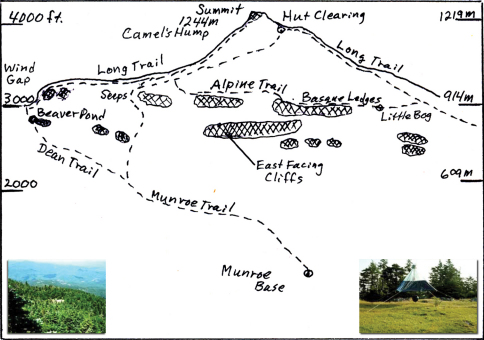
**Figure 1.** Camel’s Hump seen from the east. The Monroe Trail ascends to the cliffs and passes seeps, then crosses the Alpine Trail and ends at the Hut Clearing. The Long Trail follows the ridge line and goes up over the summit. The Dean Trail branches from the Monroe Trail and passes the Beaver Pond and reaches the Wind Gap nearby. The Little Bog is toward the northern end of the less-traveled Alpine Trail which provides access to some open rocky treeless areas (Basque Ledges) above the east cliffs and is a bad weather bypass around the summit. The Seeps may provide limited habitat for certain black flies (Simuliidae). Lower right inset shows the canopy trap in Hut Clearing as Ross Bell saw it in 1972. Lower left is a view of the Hut Clearing with canopy trap seen from near the summit with I-89 about 11 km away and no evidence of cliffs from here.

**Figure:**
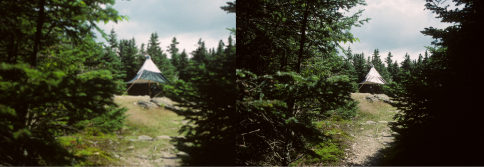
**Figure 2.** Canopy trap in Hut Clearing. Elevation 1158 m. Lower black part of trap changes bright reflection at right to a more “flat” black at left with a 90 degree turn of polarizing filter on camera lens. Photo here is from opening as Long Trail enters from the north. The Hut Clearing is a four-way junction of trails on Camel’s Hump and a convenient place for a trap, fully visible to flies.

The Tabanidae were readily identified in keys by Pechuman (1981) or Teskey (1990).

With many male and female Muscidae and Anthomyiidae in batches from the canopy trap the determining to species was a major task and must continue. The specialists working with Anthomyiidae are now deceased and revised keys to species by Griffiths (2004 and earlier) are difficult to obtain and lack a current key to genera.

The key to genera for Anthomyiidae in the Manual of Nearctic Diptera (MND) (Huckett, 1987 in McAlpine 1987) can be used but cautiously due to changes. I used the key in the MND for Muscidae supplemented by visits to collections and consulting Dr. Jade Savage.

The large quantities of muscids and anthomyiids actually pinned or pointed in 1972 were a sampling out of hundreds that were discarded. For Simuliidae the keys provided by Adler et al. (2004) were very helpful. Identification of Muscidae and Anthomyiidae involves careful observations on chaetotaxy and mistakes are easily made. I did not prepare any specimens for use of genitalia for identification. Keys to Muscidae by Huckett (1965) included some emphasis on genitalia and could enable attempts to determine species.

A Berlese-Tulgren funnel set up in the laboratory at University of Vermont allowed extracting tabanid larvae from habitat material from the Little Bog. Identification to species could be done with Teskey’s (1969) key to larvae. For maggots and puparia of Muscidae, once found, Skidmore’s (1985) book might help. Rearing maggots to adults could allow determination to species. In the late 1990’s a Special Use Permit (SUP) was required. In 2010 the SUP required a $50 fee payable to the Department of Forests, Parks, and Recreation.

An Olympus SZ stereozoom microscope worked well on specimens with an AO fiber optics high intensity illuminator. Markings on some adult Muscidae and Anthomyiidae can appear differently depending on direction viewed and kind of lighting. A 14× Hastings Triplet hand lens works with most Tabanidae and some previewing and checking of calyptrate muscoid flies.

## Results

### Canopy trap and net sweeps

Table 1 summarizes samples collected by canopy trap and net sweeps mostly from the Hut Clearing where hiking trails intersect on Camel’s Hump. In these collections both males and females were evident. Calliphorids were much more common on the summit or near feces. Tachinids were collected more with sweeps. Anthomyiids were present in most trap samples but muscids dominated. Identification of Muscidae to genus is difficult. Determining species is more difficult. While (Huckett (1972, 1974, 1977) listed species that he found on Mt. Katahdin in Maine, several high mountains in the Smokies, and the Presidential Range in New Hampshire, his keys to species of Muscidae (1965) are daunting. I present what I am able to do so that other work might follow. Comparisons with determined specimens can help. On Camel’s Hump one might find parallels with Mt. Katahdin and Presidential Range lists. Experience with some Muscidae showed, for example, that certain markings seen from above appeared black, especially with a high intensity light, but when seen from the rear they appeared white suggesting that the “lay” of the microtomentum or fine hairs affected the appearance.

**Table 1.** Diptera families in collections on Camel’s Hump, Vermont between 1972 and 2010. Emphasis on the Hut Clearing, separated by male and female. Elevation in meters, locations, elevation, method and batches (011, 9809, 1004), species, and discards (NP). CT = canopy trap; Sw = net sweeps; GM = Gressitt Malaise trap; Cmb = combined batches; St = *Stonemyia tranquilla*; Hm = *Hybomitra microcephala*; Ht = *Hybomitra pechumani*; Cm = *Chrysops mitis*. NP = Not pinned. Estimated number discarded only as mentioned in 1972 notes (Freeman 1973).

		Meter							
Date	Location	Elev	Trap/SW	TAB	MUSC	ANTH	TACH	CAL	NP
7/11/1972	BvrPnd	853	CT, 011	1Cm	0	0	0	0	128
7/13/1998	FrnGlade	1036	CT 9805	0	5♂13♀	3♂21♀	0	0	0
7/15/2010	AlpnTr	975	GM,1004	2St	5♂5♀	0	6♂4♀	2	0
7/15/2010	MnroTr	853	SW, 1002	0	0♂10♀	0	0	0	0
7/17/1972	HutClg	1158	CT, 018	3Hm	0	0	0	0	many
7/18/1972	HutClg	1158	CT018,030	4 Hp	12♂16♀	3♂20♀	2♂8♀	0	226
	HutClg		O23, Cmb						
7/21/1972	HutClg	1158	CT,9906	3Hm	4♂16♀	5♀	0♂1♀	3♀	0
7/21/1999	HutClg	1158	Sw,9914	0	0♂ 2♀	2♂6♀	0	0	0
7/21/1999	HutClg	1158	Sw,9907	3Hm ♀	5♂15♀	0♂4♀	0	0	0
7/21/1999	HutClg	1158	Sw,9909	1Hm♀	4♂8♀	5♂13♀	0	1	0
7/22/1998	FrnGlade	1036	CT,9809	0	14♂ 16♀	4♂19♀	0	0	0
7/25/1972	HutClg	1158	CT,038	0	12♂ 30♀	12♂30♀	0	0	365
8/2/1972	HutClg	1158	Sw,052	0	14♂38♀	0	0	0	0
8/3/1998	HutClg	1158	Sw,9813	0	1♂8♀	0♂2♀	1♂0♀	4	0
8/3/1972	BvrPnd	853	CT	0	12♂8♀	9♂8♀	0	0	0
8/9/1972	HutClg	1158	SW 052	0	11♂ 35♀	0	0	0	0
Total					101♂220♀	28♂121♀	9♂13♀		

Larvae of *Chrysops lateralis* were collected from habitat material from the Little Bog. This species is collected here by net sweeps rather than by trap. (No Cl in Table 1) Hikers experience attacks from deer flies when leaving the Monroe Base. The two kinds of black flies, “buzzers” and “biters”, are part of life on the trails of Camel’s Hump. Biters leave their red marks on the skin of legs, arms and neck. The buzzers near the head are more easily collected with a net than biters and gain our attention.

### Annotated list of species found on Camel’s Hump, Duxbury, Vermont

SIMULIIDAE, Black flies (Adler et al. 2004)

*Prosimulium mixtum* Syme & Davies, 1958, Species complex. Common near a seep and stream at 854 m, Monroe Trail; 011 Beaver Pond July 11, 1972; 048 Hut Clearing 1158 m, Aug 2, 1972; 033, Hut Clearing 1158 m, canopy trap, July 25, 1972

*Simulium parnassum* Malloch, 1914. 014, East side of summit, 1189 m, July 17, 1972

CULICIDAE, Mosquitoes. Small numbers on Camel’s Hump. http://www.vermontagriculture.com/ARMES/plantindustry/entomology/mosquito/MosquitoControl.html

*Aedes vexans* (Meigen, 1830) July 13, 1998

*Ochlerotatus provocans* (Walker, 1848) July 25, 1972

BIBIONIDAE

*Bibio femoratus* Wiedemann, 1820. Summit 1244 m, collected by HP Wimmer, 1972.

TABANIDAE, Deer flies and horse flies. (Burger 1995)

*Stonemyia tranquilla* (Osten Sacken, 1875) Caught by trap. Male on summit by net. Trap at Hut Clearing and Monroe Base. Non-biting small brown tabanid. Found as “*Pangonia*” in Sprague (1870).

*Chrysops carbonarius* Walker, 1848. Unknown other person. July 2, 1972. June species.

*Chrysops geminatus* Wiedemann, 1828.By net at Monroe Base 462 m; Monroe Trail 853 m, Hut Clearing 1158 m as females attacking person. Male on summit.

*Chrysops lateralis* Wiedemann, 1828. Monroe Base, Alpine Trail 945 m, Hut Clearing. Larva at Little Bog.

*Chrysops mitis* Osten Sacken, 1875. Mainly a June species, black coloration, by net at Beaver Pond, 853 m on Dean Trail in July.

*Chrysops sordidus* Osten Sacken, 1875. Netted by classmate “on Camels Hump”, has label with “Bolton, VT” but no elevation given. Found also on Mt. Mansfield (Boone et al. 2000).

*Hybomitra microcephala* (Osten Sacken, 1876). By trap. Monroe Base and Hut Clearing. Male photographed waiting on summit rock. By net sweeps at Hut Clearing. Larvae in well rotted log on wooded hillside (Nielsen 1990)

*Hybomitra pechumani* Teskey & Thomas, 1979. Determined in 1972 as *Hybomitra typhus* (Whitney, 1904) *Hybomitra pechumani* was described when *Hybomitra typhus* became 2 species. Collected by net sweeps and trap at Hut Clearing, 1158 m.

LONCHOPTERIDAE, *Lonchoptera furcata* (Fallén, 1823) 038 July 25, 1972. Hut Clearing. 1158 m.

PHORIDAE, Humpbacked flies. Smaller than a black fly.

*Megaselia pulicaria* (Fallén, 1823) 035. July 25, 1972. Little Bog 945 m.

SYRPHIDAE, Hover flies. Boone et al. (2000) listed 24 species of Syrphidae on Mt. Mansfield.

*Sericomyia militaris* Walker, 1849

SEPSIDAE, Black scavenger flies

*Sepsis punctum* (Fabricius, 1794) Known for wing waving. Found near larval habitat such as feces or decaying plant/animal material.

ANTHOMYIIDAE, Flower flies, Root Maggot Flies

*Leucophora (Proboscimyia) brevis* (Huckett, 1940)

*Delia platura* (Meigen, 1826) Seed corn maggot. Genus includes economically important anthomyiids.

*Hylemya alcathoe* (Walker, 1849)

MUSCIDAE, “House flies”

*Hydrotaea ponti* Vockeroth, 1995 Sweat fly, shiny black

*Hydrotaea militaris* (Meigen, 1826) Sweat fly, shiny black.

*Thricops albibasalis* (Zetterstedt, 1849)

*Thricops spiniger* (Stein, 1904) Very common near or above tree line (Savage 2003)

*Musca autumnalis* de Geer, 1776. Very occasional but distinctively marked.

*Helina* sp. 1 Robineau-Desvoidy, 1830

*Helina* sp. 2 Robineau-Desvoidy, 1830

OESTRIDAE, Bot flies

*Cephenemya phobifer* (Clark, 1815). Deer nasal or pharyngeal bot fly. Hovering over summit rocks, July 7 to October 21, 1245 m.

*Gasterophilus intestinalis* (DeGeer,1776) Collected on summit while hovering.

CALLIPHORIDAE, Blow flies or bottle flies

*Calliphora vomitoria* (Linnaeus, 1758)

*Lucillia illustris* Meigen, 1826. Feces, Monroe Trail, July 22, 1998

TACHINIDAE, Parasitic Flies (Larvaevoridae) www.nadsdiptera.org/Tach/home.htm Collected in the Hut Clearing, 1210 m.

*Lixophaga unicolor* (Smith, 1917), Most numerous. No host data. (NHD)

*Panzeria platycarina* (Tothill, 1921) NHD

*Billaea trivittata* (Curran, 1929) Hosts include Cerambycidae, long-horned beetles. (Arnaud 1978)

*Eulasiona comstocki* Townsend,1892 Hosts include certain leaf tier (Oecophorid) moths.

*Periscepsia (Ramonda) clesides* (Walker, 1849) NHD

*Gymnosoma par* Walker, 1849 NHD

*Oswaldia* sp. Undescribed species. Parasitoids of Lepidoptera (Arnaud 1978).

### Ecological observations

Several aspects of human ecology on Camels Hump are that people prefer the summit, they stay on trails, they are available to biting flies but sometimes make use of off trail areas by leaving feces. When people defecate in the woods they commonly do not bury their feces and they leave toilet paper. Later feces can be attractive to blow flies (Calliphoridae). Deer flies (Tabanidae) and black flies (Simuliidae) find potential hosts along trails. Since there is no overnight camping allowed currently on Camel’s Hump above 609 m, hiker activity appears on the summit around 9 AM or about two hours after people start from trail heads. Warmed and sweaty hikers can be attractive to certain sweat-loving “person flies” or “sweat flies” (*Hydrotaea militaris* and *Hydrotaea ponti*, Muscidae).

Cliffs are sunning places for Diptera (Lindner, 1973) and rocky summits provide rendezvous sites for males and females of certain flies.

Primary production mainly is the balsam fir (*Abies balsamea*), red spruce (*Picea rubens*), mountain paper birch (*Betula cordifolia*), moss mats and spinulose wood ferns (*Dryopteris spinulosa*) of the boreal forest. Sugar maple (*Acer saccharum*), yellow birch (*Betula alleghaniensis*) and American beech (*Fagus grandifolia*) occur in the lower northern hardwood forest. One large fern patch (0.1 hectare) just above the junction of the Alpine Trail with the Monroe Trail yielded anthomyiid flies in the trap. Certain species can affect ferns (Griffiths 2004, genus *Chirosia* Rondani, 1856)

Siccama (1974) emphasized defining the plant communities or forest formations on the west slope off the Burrows Trail and the changes that have occurred there were shown by Beckage et al. (2008). That there can be over 66% greater precipitation due to fog drip applies to the upper boreal and sub alpine forest (Vogelmann et al. 1968). The increased level of moisture in the sub alpine forest might relate to the abundance of Diptera. Trail wear has widened trails on Camel’s Hump and this favors flyways for Diptera. The east side of Camels Hump has a range of east-facing cliffs that are not easily seen from the summit (Fig. 1, lower left). The range of cliffs along the south and east side of the summit and below the Alpine Trail (Fig. 1, cross hatched) resulted from glacial plucking (Benitez 2004). The evidence indicates the movement of continental glaciation was from northwest to southeast (Stewart 1961, Stewart and MacClintock 1969). Hikers might see some cliffs from the Monroe Trail were it not for leaf cover above but more especially the steep bare rock under foot needing close attention. East-facing cliffs can be seen on Mt. Mansfield (1339 m) above the Cliff House, on Mt. Monadnock near Jaffrey, NH (964 m) as well as Camel’s Hump (1244 m). With the onset of rain or cloud fog the alpine and sub alpine zones change quickly. Fly activity ceases, collectors’ nets do not fly, and sounds are distorted and muffled. Eyeglasses and clothing become totally covered with droplets of fog water and trail rocks become slippery. With the threat of lightning in summer it is best to leave. People and flies do much better in sun. Flies, however, are especially adapted to take advantage of brief opportunities when sun shines and they can be seen and heard flying quickly from place to place. Lindner (1973) described the common sunning behavior of flies in the Alps. Mani (1962) emphasized aspects of microclimate on and above rocks in the Himalayas.

There is an observed preference by people to look up at the summit, to keep on trying to get there, and to enjoy the view. Biologists, however, think about where they might not yet have collected or explored for flies on Camel’s Hump and to go there. This might be a fair weather bias and a time of day bias. Collecting in fog or rain, however, is not productive.

## Discussion

Species presented here came from the east side of Camel’s Hump and the summit. The work of Boone et al. (2000, 1992) emphasized road-accessible west side stations on Mt. Mansfield (44°31'N, 72°49'W) and weekly visits. Siccama’s (1974) work and 40 years later that of Beckage et al. (2007) emphasized the west slope research area of Camel’s Hump. Open areas suitable for the canopy trap occur on the east side. Specimens available for this study survived 38 years of storage and three household moves. The future of this material is uncertain.

(Huckett (1974, 1977) listed species for Mt. Katahdin and one for the Presidential Range of Anthomyiidae and Muscidae as a reference. An analysis showed that for Anthomyiidae there were 33 species found only on Mt. Katahdin and 25 that were found only on the Presidential Range but 32 species found on both. For Muscidae there were 36 from only Mt. Katahdin, 40 from only the Presidential Range and 70 species found on both. Boone et al. (2000) listed 12 species of Muscidae but left 20 others as genus only. Of the 25 families of Diptera which they listed, Anthomyiidae, Sepsidae and Simuliidae were not included. Separations as male and female showed both sexes to be present in canopy trap collections. Flies in Tachinidae were a major component of water pan trapping on Mt. Mansfield (Boone et al. 1992). Their species list (1992) from water pan traps had few species in common with Camel’s Hump caught by net sweeps. For now, however, simply finding what flies are there is more important than comparing means of capture. The much greater moisture levels reported by Vogelmann et al. (1968) due to fog or cloud precipitation, especially nearest to trees, could keep larval habitats more moist than in lowland habitats. The Hut Clearing was about 86 vertical meters below the summit and the summit was well traveled by hikers and some with dogs. The more exposed summit experienced more wind and did not lend itself to placing the canopy trap there.

Working with flies in Anthomyiidae involved several approaches. Visiting two nearby collections (New York State Museum, Albany; University of Massachusetts at Amherst) allowed seeing specimens of species found on Mt. Katahdin and the Presidential Range. The key to genus in the MND (Huckett, 1987) for Anthomyiidae is said to be not reliable. *Delia platura*, the seed corn maggot, is abundantly present on both Mt. Katahdin ( 44♂, 18♀) and the Presidential Range (266♂, 282♀) as adults (Huckett 1972, 1977). Abundance in the Smokies (532♂, 365♀) was even greater than Katahdin or the Presidential Range (Huckett 1974). In the Diptera Catalog (Stone et al. 1965) *Delia* stands as a subgenus under *Hylemya*. H. C. Huckett states in a note there, “The subgenus *Delia* contains most of the economically important species of *Hylemya*. *Hylemya antiqua*, the onion maggot, *Hylemya brassicae*, the radish maggot, and *Hylemya platura*, the seed-corn maggot (heretofore commonly known as *cilicrura*), all cause primary injury to plant tissue above or below ground.” One must become aware of name changes at the level of species and genus as well as higher taxa. Several species of Anthomyiidae occurred with Muscidae in trap samples in July. Both males and females were found in samples. Taxonomy of Anthomyiidae today relies on Griffiths (2004) and other revisions by Griffiths, genus by genus. Currently the works of Griffiths are listed inside the cover of No. 15 showing 1–15 covering 2547 pages. Pinned Anthomyiid material used by Griffiths from the USNMNH remains in Edmonton, Alberta where he worked (Thompson pers. comm.).

Locating and identifying larvae of *Chrysops lateralis* in the Little Bog suggests that future habitat sampling of some sort might help to learn where and how the abundance of flies in Muscidae and Anthomyiidae comes from. Teskey (1990) had found larvae of *Hylemya pechumani* in wet moss and *Hylemya microcephala* in a well rotted log. The result of Skidmore’s (1985) patient documentation of hundreds of species of Muscidae allows recognition of many larvae and puparia. His frequent reference to rearing adult flies from pats of cow dung raises questions about larval habitats in the boreal forest in the absence of cows though there may be other mammals present. Decomposing local vegetation or fungi are options. Some Muscidae larvae prey on other larvae. The deer nasal bot fly (*Chrysops phobifer*) was only found hovering over the summit. Blow flies (Calliphoridae) were collected either on the summit or near dog or human feces and seldom in the canopy trap. Some dog feces can be recognized by the corn meal. Human feces generally are accompanied by toilet paper. Black flies are a background nuisance presence while checking on some loud buzzing of other flies. We looked for blow flies and they dodge and escape netting attempts readily.

The keys by Whitworth (2006) for Calliphoridae enable a good start on identification. The keys provided by Adler et al. (2004) provide better determinations to species of black flies than earlier ones but obtaining micro preparations of male genitalia may be needed. This reference has many good illustrations.

The canopy trap provided many calyptrate muscoid flies for sorting and pinning a representative sample. Most lowland trap collections had mostly tabanids and few muscoid flies. This canopy trap lasted 30 years until disposal in 2001. The bias established by the phrase “summer is July” resulted mostly from scheduling of Ross Bell’s course but takes advantage of abundance of many insects at the height of summer at higher elevations. Conditions on the mountain change in August. Collecting in June will help to confirm seasonal succession already known for tabanids. Future lists of Muscidae, Anthomyiidae and Tachinidae may lead to establishing their seasonal succession. Both *Chrysops carbonarius* and the black species *Chrysops mitis* are common spring deer fly species but can hold over into July. Significant adaptive advantages of calyptrate muscoid flies with fewer larval instars (four) than tabanids (seven) are small size, overwintering as larvae, the power of flight, ability to lay hundreds of eggs or larvae (Tachinidae) in very specific locations or on hosts may help to account for the large numbers on Camel’s Hump in the short summer.

The nose bots or deer nasal bot flies collected over the years mostly have the hair on the thorax worn down. The very first couplet in the key to species uses the nearly solid yellow hair of the thorax with a black patch over each wing base to separate *Chrysops phobifer* from the other Nearctic species (Bennett and Sabrosky 1962). The hovering behavior observed uses considerable energy and raises the question of where such energy comes from since such bot flies are known for their “vestigial” mouthparts. Do these flies derive all their nourishment from the host of the larva or might they have reduced minimal mouthparts? They can be seen hovering on visits from late July to October over the summit. Mark, release and recapture experiments might shed more light on adult longevity.

Visits to the summit of Camel’s Hump often involve interacting with people and this can detract from field time. The summit presents other problems, some related to wind, that make use of the canopy trap less attractive than, for example, the Hut Clearing.

Setting up and leaving the labeled canopy trap in the Hut Clearing was far more practical and was a more sheltered place. Careful observation of *Hybomitra microcephala* waiting on summit rocks or hovering of the bot flies, however, was more productive than use of a trap. The observer must be there to see and photograph them.

*Hydrotaea militaris* and the less common *Hydrotaea ponti* are of personal interest because they are obvious bothersome 5 mm shiny black muscid sweat flies that do not bite.

Hovering and waiting are behaviors common to male tabanids. But *Hydrotaea microcephala* and *Chrysops geminatus* show waiting behavior on this summit as they do at Mt. Rigaud west of Montreal (Leprince et al. 1983). Wilkerson et al. (1985) summarized hovering and swarming for many tabanid species.

Changes in the plant communities on Camel’s Hump have been found by Beckage et al. (2008). They recorded increases in monthly mean temperature, total annual precipitation, length of growing season, winter mean temperature and total winter precipitation. It will be a long time before we are able to define or assign changes in the invertebrate communities. They found changes between 1965 and 2005 by revisiting where Siccama (1974) worked and consulting continuing weather records.

An older map of Camel’s Hump (Sawyer 1990) shows that much of the study area had been influenced by a fire in 1903 on both the east side and northwest of the summit as well. Such a disturbance plus environmental conditions present in the boreal, sub alpine and alpine areas encourage more birch. This map shows the location of the “CHClub Hut”. Even today pieces of an iron stove can still be found just east of the present Hut Clearing, the location of many canopy trap setups. Sprague (1870) may have used the carriage road.

### Polarized light

Polarized skylight refers to the band of maximum polarization of light overhead at 90 degrees to the sun. Reflections from water, honeydew, or the canopy trap may also affect flies. Polarized light is part of the sensory ecology of invertebrates. With land sloping away on this mountain there is a maximum view of the eastern sky. How it influences flies here remains to be determined.

In mid morning the band of polarized light would sink behind the summit in the west and northwest for sites on the east slope of Camel’s Hump above 762 m. Observing fly behavior with polarized light in mind is one more aspect of their ecology. Wellington (1974) noted that this band of maximum polarization is visible 2 hours before and after sunset and sunrise and affects the behavior of mosquitoes. Domestic hive bees have hairy compound eyes and are known to use polarized light as one of several environmental cues for navigation (Frisch 1949, others since). Blow flies (Wunderer and Smola 1982) and several other invertebrates somehow use polarized light as do fish (Hawryshyn 1992; Land 1991) and birds (Able and Able 1993). Wada (1974) found special ommatidia in the dorsal and dorsolateral marginal areas of compound eyes of both sexes in 29 species in 13 families of Diptera including Tabanidae, Muscidae and Calliphoridae.

More specifically Strausfeld and Wunderer (1985) traced axons from receptor cells in ommatidia within the dorsal marginal region of *Calliphora erythrocephala* to the medulla of the brain. They provided electrophysiological evidence of sensitivity to polarized skylight which could add celestial navigation to the use of landmarks and olfactory signals. Various male flies have enlarged dorsal ommatidia and exhibit holoptic compound eyes quite different from more widely spaced dichoptic compound eye of females. Certain muscid flies caught on Camel’s Hump have very hairy eyes. People notice water, a colorless and clear liquid, on a kitchen floor or other surface due to the reflection of polarized light. Indeed, when it rains the reflections of sky ight, now polarized, are everywhere. With rain, nearly all fly activity shuts down on Camel’s Hump. Aerial nets don’t work, there is no sunshine, and trail rocks become slippery, as conditions change for both flies and people. We are able to observe how objects like a canopy trap might appear by rotating a polarizing filter 90 degrees on a camera or turning polarizing sun glasses similarly (Fig. 2). Hunter and Ossowski (1999) found sugars from honeydew in crops of female horse flies wild-caught by net sweeps. This suggests a way for flies to obtain a source of flight energy in a place known for fog, rain and pitch. What do those calyptrate muscoid flies use for their flight energy? Is there, perhaps, a source via honeydew from aphids or leafhoppers in tree crowns?

Change of appearance of the canopy trap in the Hut Clearing can be seen in Fig. 2. How flies might process this visual difference remains to be explored experimentally.

Whatever light reaches the insect compound eye might otherwise be reflected except for structural features preventing reflection. Miller et al. (1968) found surface features called corneal nipples on the outer surface of ommatidia or facets of compound eyes of monarch butterflies and a common horse fly. Corneal nipples can reduce reflection “by 1000-fold”. Facets on ommatidia on the upper half of the holoptic compound eye of many male dipterans can be twice the diameter of those on the lower portion. But not all males of Muscidae show this, nor do all tabanids. Wilkerson et al. (1985) included diameters ranging from 100 percent larger down to 30 percent larger. Mazokhin-Porschniakov (1969) provided a listing of arthropods known to respond to polarized light.

### Taxonomic impediment

The designation of “taxonomic impediment” refers to the lack of taxonomic expertise in research on biodiversity and ecology. Declining numbers of taxonomists and increasing demand for identifications have had their effect on this study. Workers with tabanids have had access to help from several taxonomists. This directly affects our ability to list species as found in new places and results in unevenness in representation on checklists. Boone et al. (2000) did not include Anthomyiidae, Sepsidae or Simuliidae in their Checklist for Mt. Mansfield State Park in Vermont. Determined specimens are the taxonomic infrastructure needed as reference for future ecological work.

The difficulty in determining species in Muscidae and Anthomyiidae from adult specimens leaves this study with many undetermined specimens. That Skidmore (1985) used reared larvae and puparia of Muscidae allows us to approach some Muscidae through their larval habitat. He often refers to cow pats but Camel’s Hump features the moist sub alpine and boreal forest with certain small bogs as potential habitat but no cows. Separating or extracting larvae of Diptera from habitat samples and rearing such larvae to adults can sharpen our focus on the role of species living on Camel’s Hump.

The much closer look at larvae and puparia of Muscidae by Skidmore (1985) notes the rather common occurrence of viviparity in Muscini and Reinwardtinae within Muscidae, a strategy most often associated with Tachinidae (Larvaevoridae). Calyptrate muscoid flies already exhibit only four larval instars with the fourth one normally encased in the larval skin of the third instar. By passing the first larval instar in the oviduct of the female fly this puts part of the burden of nourishment on the adult female fly. This reduces the need for larval foraging for food. Given sufficiently sun-warmed conditions a female fly can exploit limited food and habitat opportunities more efficiently.

## Conclusions

First, what Ross Bell started must continue as we build on the basis begun here. Second, the huge quantities of Muscidae and Anthomyiidae emphasize that the nature of larval habitats is yet to be determined. Third, further more targeted collecting is needed, especially of spring species of Tabanidae, in June on Camel’s Hump. Fourth, the relation to flies of east-facing cliffs of the summit and cliffs at elevation 850 m below the Alpine Trail needs further study. Fifth, as it becomes available after completion of building renovations, the collection of Muscidae at Bishop’s University deserves a visit and further study. Sixth, revised keys to Anthomyiidae should continue to be improved to continue the work of GCG Griffiths.

Huckett’s (1965) keys to species of northern Muscidae were done when Anthomyiidae was included as a subfamily of Muscidae. Access to “known’s” in various collections did, however, include species determined by HC Huckett. Revised keys to genus for Muscidae and Anthomyiidae are a major need.

## Appendix 1

### Canopy trap details

A canopy trap is composed of 182 cm equilateral triangular faces with upper half as clear “6 mil” (0.15 mm) plastic and the lower half of “6 mil” black plastic. A pipe clamp holds all four sides onto the base of an outer 15 cm diameter galvanized sheet metal sleeve. Another slightly smaller cylindrical sleeve must slide into the lower sleeve and have above it a tilted cone of aluminum mosquito screen that leads into a 15 cm powder funnel firmly supported by a bracket with side braces. The screen funnel seam can be sewn with pulled out aluminum wire and sealed to funnel with silicone adhesive. The bracket has the lid of the collecting jar bolted below. Other attachments involve drilling holes and peening over rivets made from aluminum tacks or using pop rivets.

A Mason jar can then be fitted with the killing agent. Radiating hem lines are stitched with durable fabric and a brass grommet is placed at each corner. The lower black portion can be lined with fabric to maintain strength under the tension of tie-downs. Midway down in the top sleeve four sets of drilled holes allow installing wires attached to a central circle of metal (“spider”) to allow the spike in top of support pole to hold trap at desired height. Plastic tent pegs allow staking the trap out so as to keep the canopy approximately level. A plastic beach ball spray painted with glossy black enamel and inflated each time can hang below the trap. When flies pass under the trap they tend to buzz upward to find a way out. Eventually they reach the leaning screen cone and the powder funnel where light comes through and the fumes from either calcium cyanide or DDVP (Dichlorvos 18.6%, No-Pest Strip) causes them to die. Sometimes clear plastic wrap will work as a cover. Either a bag of dry ice or a small vial with a pipe cleaner wick charged with Octenol will enhance the catch. A canopy trap apparently provides a producer of heat from black plastic in sunshine, a sharp and non-plant outline, and reflection of polarized light like hair on a large mammal. With carbon dioxide it imitates exhaled breath of a large mammal. This trap generally catches more horse flies than deer flies and has served as a good survey tool. This trap allows the collector to go elsewhere catching deer flies or other flies with an aerial net.
